# A Simple, Organic Solvent-Free, and Scalable Process for Producing Calcium Alginate Nanoparticle Powder by High-Pressure Homogenization and Spray Drying

**DOI:** 10.1021/acsomega.6c01781

**Published:** 2026-06-29

**Authors:** Paulo Augusto Marques Chagas, João Otávio Donizette Malafatti, Lívia Rodrigues Boueri de Souza, Maria Sirlene Morais, Gabriela Fávero Galvão, Guilherme Alves Pinto, Luiz Henrique Capparelli Mattoso, Wanderley Pereira de Oliveira

**Affiliations:** † Faculty of Pharmaceutical Sciences of Ribeirão Preto, University of São Paulo (FCFRP USP), Ribeirão Preto, SP CEP 14040-903, Brazil; ‡ National Laboratory for Nanotechnology for Agriculture (LNNA), Embrapa Instrumentação, Rua XV de Novembro 1452, Centro, São Carlos, SP 13561-206, Brazil

## Abstract

Scaling up nanoparticle production remains one of the main bottlenecks for translating laboratory formulations into industrially relevant processes. This study established an organic solvent-free and reproducible process to produce calcium alginate nanoparticles by integrating ionic gelation, high-pressure homogenization, and spray drying. Scale-up of the synthesis step was demonstrated by increasing the ionic gelation batch volume from 400 mL to 2 L, while high-pressure homogenization at 1000 bar was subsequently applied as a refining step, reducing particle size from the micrometer range to approximately 223 nm at 400 mL scale and 236 nm at 2 L scale. The spray-dried powders exhibited low water activity, stable morphology, and a predominantly amorphous structure, as indicated by SEM, TEM, XRD, XRF, and thermal analyses. The integrated workflow yielded nanoparticles with structural integrity and controlled physicochemical properties, supporting the potential of this platform for future studies involving encapsulation systems and related technological applications.

## Graphical abstract

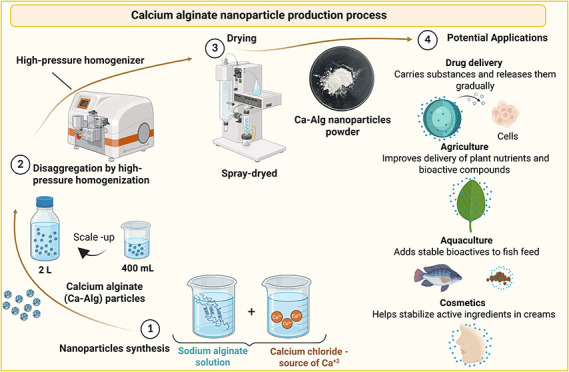

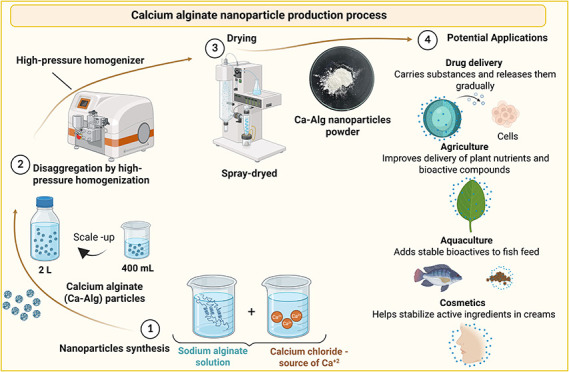

## 1 Introduction

Nanoparticles produced from natural polymers have attracted considerable interest due to their mild processing requirements and broad technological potential. Among these systems, calcium alginate nanostructures are commonly obtained by ionic gelation, in which sodium alginate interacts with calcium ions to form dispersed hydrogel-like particles under aqueous and organic solvent-free conditions.^1–5^

Although ionic gelation is effective at laboratory scale, it is most often reported for relatively small production volumes, typically in the range of a few hundred milliliters. When transferred to higher volumes, the process may become less reproducible due to broader particle size distributions, increased aggregation, and loss of colloidal uniformity, directly affecting critical quality attributes such as hydrodynamic diameter, polydispersity index, and suspension stability.^5^ Therefore, strategies capable of refining the dispersed structures after gelation are relevant for improving process control and maintaining nanoscale characteristics during scale-up.

High-pressure homogenization (HPH) has been widely used to modify the structural organization of biopolymeric systems and improve the physicochemical properties of colloidal dispersions. The intense shear, cavitation, and impact forces generated during HPH can disrupt larger aggregates and contribute to the formation of more homogeneous dispersed systems.^6^ Previous studies have also proposed alternative strategies for alginate processing. For example, Pravinata et al.^7^ described the use of the Leeds Jet Homogenizer, which relies on two colliding liquid jets, one containing sodium alginate and the other calcium chloride. To further reduce particle size and disaggregate clusters, an additional sonication step was required.^7^ While effective, this jet-collision system differs from valve-based HPH, which achieves disruption predominantly through shear and cavitation and is more suitable for scale-up. However, even when reduced aggregation and improved homogeneity of the suspension, nanosuspensions obtained by ionic gelation remain difficult to handle, store and stabilize because the high-water content accelerates aggregation, microbial growth and physicochemical degradation.

In addition to particle refinement, another important challenge is the stabilization and handling of the resulting aqueous suspensions. Even when nanoscale dispersions are obtained, their high-water content may impair storage, transport, and physicochemical stability. In this context, spray drying is an attractive downstream strategy because it enables the conversion of liquid dispersions into dry powders with lower water activity, easier handling, and potential redispersion, while also representing a continuous and scalable processing step.^8–12^

The overall process investigated in this study is summarized in Scheme 1, which illustrates the sequential stages of synthesis by ionic gelation, refinement through high-pressure homogenization, and powder recovery by spray drying. Unlike previous approaches focused only on nanoparticle formation or postgelation disaggregation, the present study proposes an integrated route in which the ionic gelation step was scaled from 400 mL to 2 L, followed by valve-based HPH as a refining step to improve particle size homogeneity and, finally, by spray drying to obtain dry material. This combination addresses a relevant gap in the processing of calcium alginate nanostructures, namely the limited availability of scalable routes that combine size refinement, increased solids content, and powder stabilization in a single workflow.

**Scheme 1:**
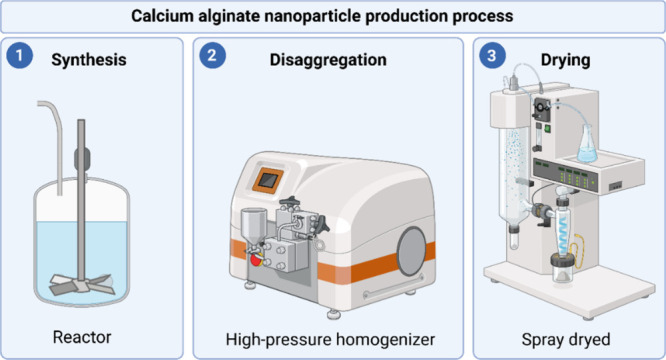
Calcium Alginate Nanoparticle Production Process Comprising Three Stages: (1) Synthesis in a Reactor by Ionic Gelation, (2) Disaggregation by High-Pressure Homogenization, and (3) Drying by Spray-Dryer^a^

a Created in BioRender. Chagas, P. (2026) https://BioRender.com/q36v641.

In this context, the present study investigated whether high-pressure homogenization could be used as an effective refining step for calcium alginate nanostructures produced by ionic gelation, with emphasis on aggregate reduction and improvement of size homogeneity after scale-up. The study also evaluated whether this strategy remained effective at higher alginate concentration and whether the refined suspension could be successfully converted into a dry and redispersible powder by spray drying. Thus, this work proposes an integrated and organic solvent-free route combining ionic gelation, high-pressure homogenization, and spray drying for the production and stabilization of calcium alginate nanostructures.

## 2 Materials and Methods

### 2.1 Chemicals and Reagents

Sodium alginate (61% mannuronic acid and 39% guluronic acid, with molecular weight in the range of 12,000–40,000 g/mol, Sigma-Aldrich, St. Louis, MO, USA), calcium chloride dihydrate (CaCl_2_·2H_2_O, Sigma-Aldrich, St. Louis, MO, USA), and reverse osmosis (RO) water were used in this study. The RO water presented conductivity of 0.31 μS·cm^–1^. It was employed for preparing alginate and calcium chloride solutions, washing the suspensions, dilution before analyses, and as the continuous phase during ionic gelation and high-pressure homogenization. All reagents were of analytical grade and used without further purification.

### 2.2 Synthesis of Calcium Alginate Nanoparticles

Calcium alginate nanoparticles were obtained by ionic gelation using sodium alginate solutions at 0.05% (w/v) and 1% (w/v) to evaluate the effect of polymer concentration on nanoparticulate formation, structural organization, and surface characteristics. The lower concentration (0.05%) was selected to ensure the formation of well-dispersed nanoscale systems,^13^ while the higher concentration (1%) was used to increase solid content and assess its influence on particle morphology and yield. Sodium alginate powder was dissolved in reverse osmosis water under magnetic stirring at room temperature for 2 h until complete dissolution. The solutions were then refrigerated for at least 24 h at 8 ± 3 °C to ensure complete polymer hydration before cross-linking. Ionic gelation was performed by dripping the sodium alginate solution (0.05 or 1% (w/v)) into a 1% (w/v) calcium chloride dihydrate solution kept under continuous stirring at 1100 rpm, with the addition controlled by a peristaltic pump operating at 1 mL/min to ensure uniform dispersion and reproducible nanoparticle formation. The addition direction, from alginate into calcium, was strictly maintained. After dripping, the suspensions were stirred for an additional hour to complete cross-linking. Two production scales were employed, with 400 mL representing the laboratory scale (LC) and 2 L representing the batch scale (B), used to investigate the scalability of the process.

### 2.3 Processing by High-Pressure Homogenization (HPH)

After the ionic gelation process, the obtained suspension was washed with RO water using three successive washes with 3-fold the sample volume to remove excess calcium chloride and unreacted ions. The washed calcium alginate suspensions were then processed by high-pressure homogenization (HPH) in a GEA Niro Soavi PandaPLUS homogenizer. The suspensions processed by HPH were subjected to three consecutive passes at 1000 bar, generating the 1C, 2C, and 3C samples, while the nonhomogenized nanosuspension was designated as 0C. These HPH-processed suspensions corresponded to the same batch volumes established before the washing step, namely 400 mL and 2 L, and were originally prepared using sodium alginate at 0.05 or 1% (w/v), in both cases with 1% (w/v) CaCl_2_ solution.

### 2.4 Spray Drying Process

Among the prepared formulations, the suspension containing 1% (w/v) sodium alginate after the third homogenization cycle (3C) was selected for spray drying, as the refined state combined with the higher polymer content favored powder recovery and provided sufficient solid yield for the subsequent analyses. The suspensions containing calcium alginate nanoparticles were dried using a Spray-Dryer SD-05 (LabPlant, Huddersfield, UK) equipped with a drying chamber of 215 mm in diameter and 500 mm in height, operating under a cocurrent flow configuration. The operational parameters were set as feed rate (*W*_susp_) of 7 mL/min, atomization pressure (*P*_atm_) of 2 kgf/cm^2^, atomization air flow rate (*W*_atm_) of 20 L/min, and nozzle diameter of 1 mm. The inlet air temperature was maintained at 150 °C, while the outlet air temperature ranged between 70 and 80 °C. The process was initiated by heating and stabilizing the drying air, followed by the continuous feeding of the nanoparticle suspension under controlled stirring. The outlet gas temperature was monitored at 10 min intervals to verify process stability throughout the spray drying. Drying yield was calculated as the ratio between the dry mass collected after spray drying and the total dry mass fed into the system.^14^ The water activity of the powders was evaluated in triplicate using an AquaLab 4TEV instrument (Decagon Devices, Pullman, WA, USA) equipped with a capacitance sensor. Moisture content of the powder was determined using a thermobalance (Sartorius MA35) at 105 °C until constant mass was reached.

### 2.5 Physicochemical Characterization

The characterization procedures were conducted in two stages to evaluate the influence of process parameters and formulation composition on the structure and properties of the calcium alginate systems. The first stage aimed to test the initial hypothesis that high-pressure homogenization (HPH) could effectively reduce the size of calcium alginate particles obtained by ionic gelation using a 0.05% (w/v) sodium alginate solution. The colloidal characteristics were analyzed by dynamic light scattering (DLS) using a Zetasizer Nano-ZS90 (Malvern, England) at 25 °C. The analysis provided the hydrodynamic diameter (Z-ave), polydispersity index (PdI), and ζ-potential (ZP) of the calcium alginate nanoparticles. Nanoparticles were prepared in three independent batches under the same experimental conditions to assess process reproducibility, and DLS measurements were performed for each obtained sample. The results are expressed as mean ± standard deviation (sd). The pH of the calcium alginate nanoparticle suspensions was measured at room temperature (25 ± 2 °C) using a calibrated Metrohm 827 pH Lab meter (Metrohm AG, Herisau, Switzerland) after each homogenization cycle.

For morphological evaluation, a 10-fold diluted dispersion was drop-cast onto aluminum foil mounted on SEM stubs with conductive carbon tape and dried at room temperature. High-resolution images were acquired using a FEI Magellan 400 L Field Emission Gun (FEG) Scanning Electron Microscope (SEM), and complementary imaging was performed on a Philips XL 30 TMP SEM. Elemental analysis and mapping were performed using an Oxford EDS system coupled to the microscope.

After confirming nanoscale particle formation and colloidal stability, the second stage was carried out using a 1% (w/v) sodium alginate solution. This phase was designed to increase the overall yield and to test the second hypothesis, which evaluated whether HPH would remain effective at higher polymer concentrations while enabling the formation of stable and homogeneous nanostructures. A comprehensive set of complementary characterizations was conducted to correlate synthesis conditions with the physicochemical and structural properties of the materials and processes.

The elemental composition of the calcium alginate spray dried powder was determined by X-ray fluorescence (XRF) using a Shimadzu EDX-720 energy-dispersive spectrometer. The crystalline phases were analyzed by X-ray diffraction (XRD) using a Bruker D8 Advance ECO diffractometer equipped with Cu Kα radiation (λ = 1.5406 Å). Powder samples were scanned in the 2θ range of 10°–90°, with a step size of 0.02° and a count time of 1 s per step. The specific surface area (SSA) and pore diameter were determined by nitrogen adsorption and desorption using the Brunauer–Emmett–Teller (BET) method with a Micromeritics Gemini VII analyzer. Prior to analysis, samples were degassed under vacuum at 80 °C for 12 h. Nitrogen adsorption–desorption isotherms were recorded at 77 K, and SSA values were calculated in the relative pressure region *P*/*P*_0_ ≤ 0.3. Pore size distribution and total pore volume were determined from the desorption branch of the isotherm using the Barrett–Joyner–Halenda (BJH) model.^15^

Thermal properties were evaluated by differential scanning calorimetry (DSC; Jade DSC, PerkinElmer, Waltham, MA, USA) and thermogravimetric analysis (TGA; TGA 4000, PerkinElmer, Waltham, MA, USA). For DSC, 5 to 10 mg of each sample was heated from 25 to 200 °C at 10 °C min^–1^ under a nitrogen flow of 30 mL/min. For TGA, approximately 10 mg of each sample was heated from 25 to 880 °C at 10 °C min^–1^ under a nitrogen flow of 20 mL/min.

The morphology and nanoscale structure were investigated by transmission electron microscopy (TEM) using a Talos Cold Field Emission Gun (Cold FEG) microscope operated at 200 kV. The samples were dispersed in ethanol, deposited on carbon-coated copper grids, and dried under ambient conditions. Bright-field images were acquired to evaluate particle size, shape, and aggregation state, and energy-dispersive X-ray spectroscopy (EDS) integrated into the microscope was used for elemental mapping and correlation between morphology and composition.

Nanoparticle Tracking Analysis (NTA) was performed using a NanoSight NS300 system (Malvern Instruments, United Kingdom) equipped with a 488 nm laser and an sCMOS camera to determine the size distribution of calcium alginate nanoparticles. The spray-dried nanoparticle powder was resuspended in ultrapure water at a concentration of 0.5 mg/mL prior to analysis. Measurements were conducted at 25 °C, and the particle size distribution was obtained from the recorded trajectories using NTA software. Additional characterization data are provided in the Supporting Information.

## 3 Results and Discussion

Alginate has been widely studied regarding its physicochemical characteristics and its application in creating microparticles and bulk hydrogels.^16–18^ It is considered nontoxic, biodegradable, inexpensive, readily sourced, mucoadhesive, biocompatible, and nonimmunogenic.^19,20^ Alginate is an anionic polysaccharide obtained from brown seaweeds and certain bacteria, composed of β-D-mannuronic acid (M) and α-L-guluronic acid (G) residues linked linearly by 1,4-glycosidic bonds. The ratio and sequence of M and G units vary according to the algal source and determine the physicochemical properties of the polymer. These structural features directly influence its ability to form gels, which typically occurs through ionic cross-linking with multivalent cations, mainly calcium (Ca^2+^), which is the preferred and most widely used ion due to its mild reactivity, biocompatibility, low toxicity, and regulatory acceptance.^21^ Other multivalent ions such as barium (Ba^2+^), strontium (Sr^2+^), zinc (Zn^2+^), or ferric (Fe^3+^) can also induce gelation, although their use is generally restricted because of cytotoxicity concerns and limited regulatory approval. During gelation, sodium ions associated with G-rich segments are replaced by Ca^2+^, and adjacent G blocks align to produce the characteristic egg-box junctions.^22^ Multiple chains are thereby interconnected to yield a three-dimensional network, often containing more than 95% water.^23^ In the present study, the sodium alginate employed contained approximately 61% mannuronic acid and 39% guluronic acid, resulting in an M/G ratio of 1.56, with an estimated molecular weight in the range of 12,000–40,000 g/mol.

In external gelation, alginate solutions are introduced as droplets into a bath containing cations, such as calcium chloride. The cations diffuse inward from the continuous phase, establishing a gelled matrix that progresses from the droplet surface toward its core, a mechanism commonly referred to as the diffusion method.^24^ Tian et al.^21^ reported that calcium alginate gels gradually release calcium in gastric and intestinal media, allowing the beads to function both as structural carriers and as sources of bioavailable calcium.

To facilitate the understanding of alginate structure and gelation mechanisms discussed above, Figure 1a–d presents the monomeric units of α-L-guluronic acid (G) and β-D-mannuronic acid (M) (a, b), the polymer chain formed by alternating residues (c), and the ionotropic gelation process described by the egg-box model (d).

**Figure 1:**
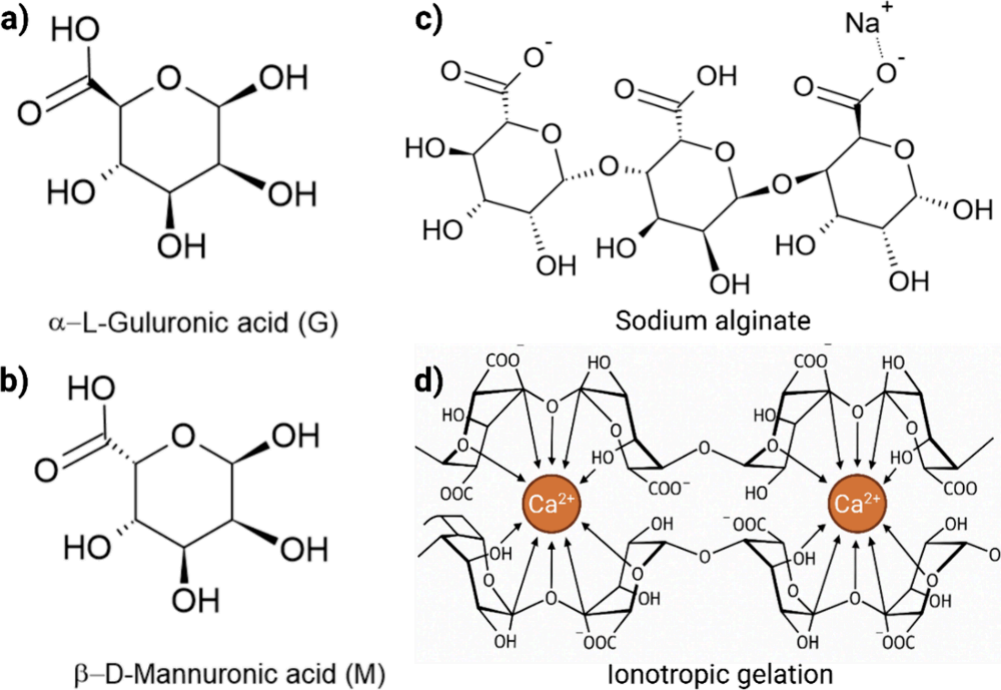
Structural representation of the alginate monomeric units: (a) α-L-guluronic acid (G) and (b) β-D-mannuronic acid (M). In (c), the sodium alginate polymer composed of alternating residues is shown, while in (d) the ionotropic gelation mechanism described by the “egg-box” model is illustrated.

Building on this structural framework, the principles governing alginate gelation were directly applied to the development of calcium alginate nanoparticles. Process parameters at different scales strongly influenced particle size, distribution, and stability. The transition from small to larger volumes was explored to understand how laboratory conditions translate into production settings.

To translate these theoretical aspects into practical results, the process was first carried out on a small scale (approximately 400 mL) using a beaker under magnetic stirring, where precise control of droplet addition and mixing promoted the formation of well-dispersed nanoparticles (Figure 2a). The synthesis was then scaled up to 2 L in a glass vessel under continuous stirring (Figure 2b), maintaining the same ionic gelation mechanism under conditions more representative of production scale.

**Figure 2:**
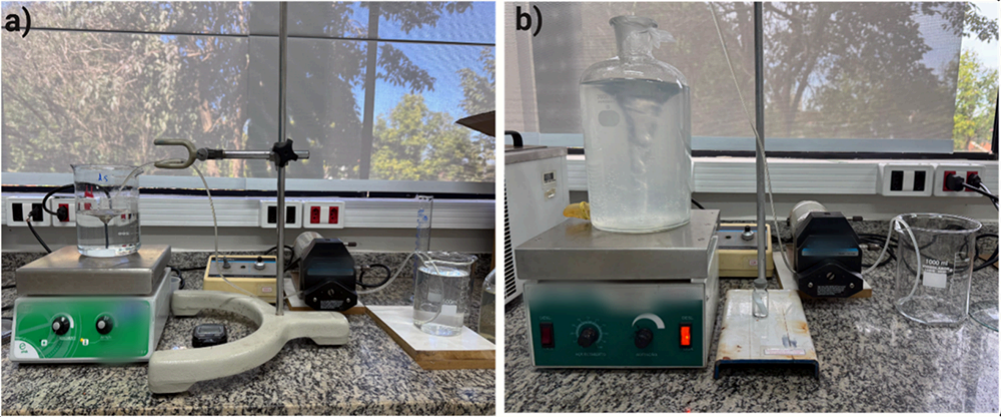
Experimental setup for calcium alginate nanoparticle synthesis by ionic gelation using a sodium alginate dispersion at 0.05% (w/v). (a) Laboratory-scale synthesis at 400 mL and (b) batch-scale synthesis at 2 L, both performed by dripping into a 1% (w/v) CaCl_2_ solution under continuous stirring.

Larger volumes, however, can modify hydrodynamics and mass transfer, potentially leading to variations in particle size, polydispersity, and surface charge. Reports in the literature confirm that alginate nanoparticle properties are highly sensitive to mixing conditions, ionic concentrations, and addition rates,^25^ which highlights the importance of reproducing these conditions consistently during scale-up.

Overall, the experimental setups demonstrated the feasibility of scaling up calcium alginate nanoparticle synthesis while maintaining control over the gelation process. This controlled transition not only validates the scalability of the ionic gelation approach but also provides a solid foundation for the subsequent refinement step by high-pressure homogenization, which enables further control of particle size and dispersion uniformity.

High-pressure homogenization (HPH) is widely employed due to its simplicity, scalability, and ability to generate uniform dispersions without the need for organic solvents or large amounts of stabilizers, which makes it suitable for food, pharmaceutical, and biopolymeric systems.^26^ It allows reproducible production of nanoparticles with narrow size distribution and high colloidal stability, which are essential for large-scale processes. As reported by Patel and Pathak,^27^ HPH is recognized as a reliable and energy-efficient method for refining nanoparticles in both lipidic and polymeric matrices. In this study, it was adopted as a postgelation refining step to enhance uniformity, stability, and reproducibility of calcium alginate nanoparticles.

Figure 3 illustrates the integrated process for producing calcium alginate nanoparticles through the combination of ionotropic gelation and high-pressure homogenization (HPH). In Figure 3a,b, sodium alginate and calcium chloride solutions are shown as precursors that react to form cross-linked Alg–Ca nanoparticles. Figure 3c schematically represents the ionotropic gelation mechanism, while Figure 3d,e depict the refinement step performed by HPH at different scales. The process was carried out under three consecutive cycles at 1000 bar, generating the samples referred to as 0C (before homogenization) and 3C (after three cycles). Figure 3d shows the results obtained at 400 mL, and Figure 3e at 2 L, where the increased transparency of the 3C suspensions indicates reduced aggregation and enhanced colloidal stability. This macroscopic evidence of size reduction, together with the absence of particle regrouping in both scales, indicates improved stability of the nanosuspensions and is further corroborated by the dynamic light scattering analyses presented below.

**Figure 3:**
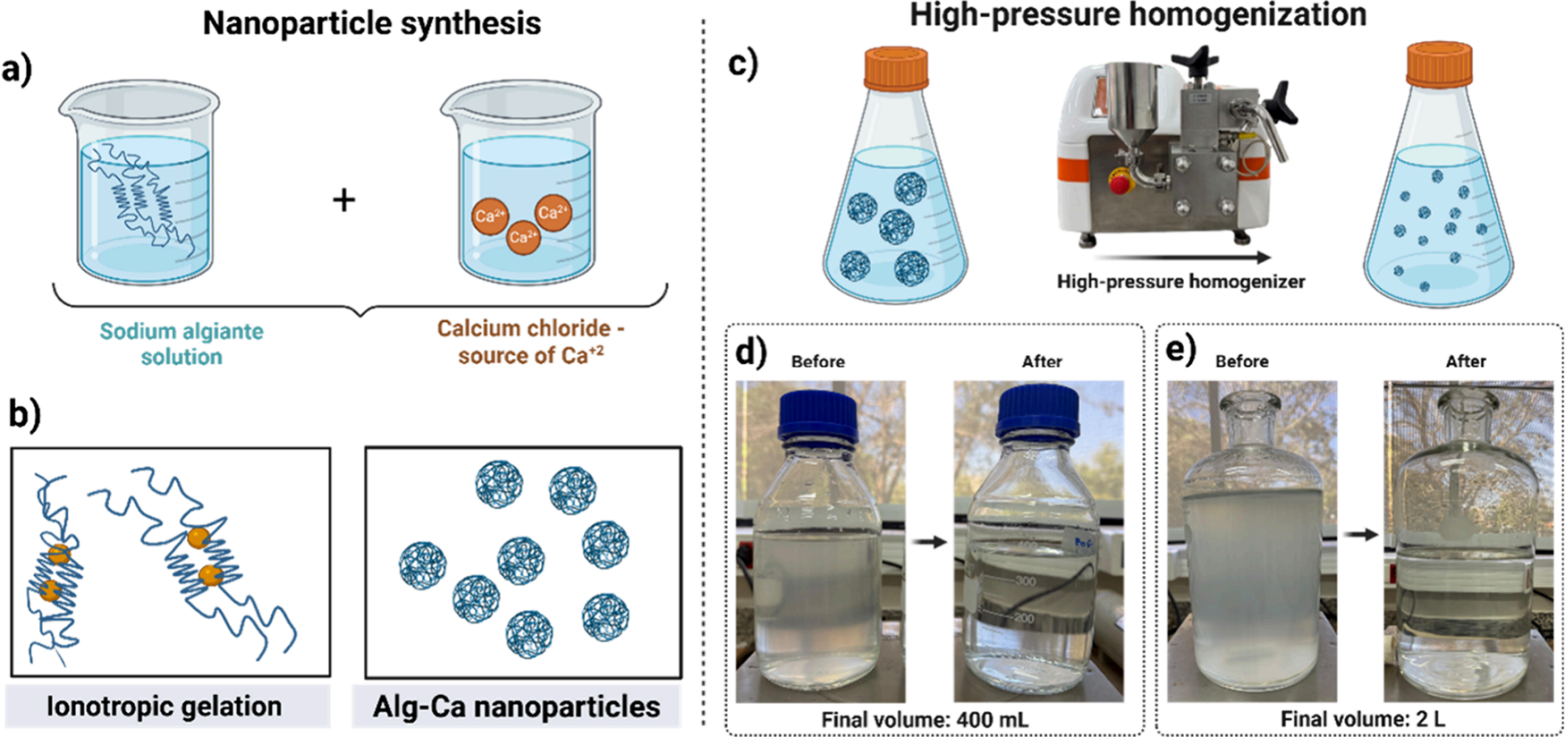
Integrated process for calcium alginate nanoparticle production combining ionotropic gelation and high-pressure homogenization (HPH). (a) Ionotropic gelation scheme. (b) Formation of Alg-Ca nanoparticles. (c) High-pressure homogenization step. (d, e) Suspensions before and after three HPH cycles at 1000 bar for final volumes of 400 mL and 2 L, respectively. Created in BioRender. Chagas, P. (2025) https://BioRender.com/h9e9sy5.

### 3.1 Physicochemical Characterization by Dynamic Light Scattering (DLS) and ζ-Potential

The mean hydrodynamic diameter, polydispersity index (PdI), and ζ-potential (ZP) are fundamental parameters for characterizing nanoparticle suspensions, as they provide information on particle size, size distribution, and surface charge, respectively. The hydrodynamic diameter represents the apparent size of nanoparticles in a liquid medium, accounting for the solvation layer surrounding each particle.

The PdI is a measure of the particle size distribution, where values below 0.3 indicate narrow and homogeneous distributions, while higher values denote heterogeneity and potential instability. The ζ-potential provides an index of surface charge density and electrostatic repulsion, and high absolute values typically promote stability by preventing aggregation through electrostatic forces.^28^

These parameters were used to evaluate the influence of high-pressure homogenization on the dispersion behavior of calcium alginate nanoparticles. In this work, the samples were designated as 0C_LC (untreated), 1C_LC, 2C_LC, and 3C_LC, corresponding to suspensions subjected to zero, one, two, and three high-pressure homogenization cycles, respectively. As shown in Figure 4a, in the 400 mL synthesis, calcium alginate nanoparticles exhibited a drastic reduction in mean size after high-pressure homogenization (HPH). The untreated suspension exhibited a diameter of 1282.0 ± 229.0 nm, reflecting intense aggregation.

**Figure 4:**
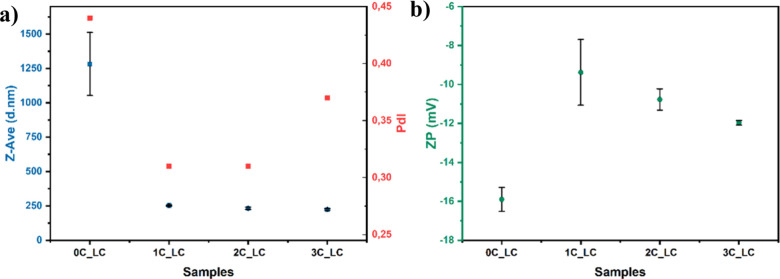
Physicochemical characterization of alginate nanoparticles subjected to different numbers of homogenization cycles (0C_LC, 1C_LC, 2C_LC, and 3C_LC): (a) hydrodynamic diameter (Z-Ave) and polydispersity index (PdI) and (b) ζ-potential (ZP). Data are presented as mean ± standard deviation.

A single homogenization cycle (1C_LC) at 1000 bar reduced the particle size to 252.9 ± 3.3 nm, and subsequent cycles further decreased the mean diameter to 232.5 ± 8.4 and 223.5 ± 5.4 nm. The strong reduction highlights the disruptive action of HPH, including shear, turbulence, and cavitation, which fragment aggregates and disperse the particles more uniformly.^7^ Comparable reductions in size and stabilization effects were also reported for soybean protein isolate and sodium alginate nanoemulsions, where HPH produced smaller droplets with improved dispersion stability.^29^

The PdI values further demonstrate the influence of high-pressure homogenization on improving particle uniformity. The untreated sample (0C_LC) exhibited a PdI of 0.44 ± 0.05, characteristic of a broad size distribution. After homogenization, values decreased to the 0.31–0.37 range, indicating a narrowing of the size distribution and improved dispersion consistency compared to the untreated sample. This indicates that HPH not only reduces average size but also disrupts irregular aggregates formed during ionic gelation, leading to a more homogeneous nanoparticle suspension. As shown in Figure 4b, the ζ-potential values shifted from −15.9 ± 0.62 mV in the untreated suspension to moderately less negative values between −9.38 and −11.97 mV after homogenization. The negative surface charge of alginate, due to carboxylate groups, provides inherent electrostatic stabilization. Accordingly, shifts in pH, ionic strength, and calcium-mediated cross-linking modulate the ζ-potential and thereby the stability of the suspension.^18^ Maintaining appropriate ζ-potential is essential for ensuring the effective performance and shelf life of alginate-based drug delivery systems.^5^

Using the same ionic gelation and high-pressure homogenization protocol applied at 400 mL, the 2 L run reproduced the expected behavior, with a pronounced reduction in hydrodynamic diameter after homogenization and a concurrent narrowing of the size distribution. These results corroborate the efficiency and reproducibility of the proposed refining step at the 2 L batch, supporting its applicability after scale-up of the ionic gelation stage. Figure 5 shows that the mean Z-Ave decreased significantly from 12,880 ± 1890 nm in the pre-HPH suspension to 296 ± 1.53 nm after one cycle (1C_B) at 1000 bar, and further to 248 ± 2.1 nm and 236 ± 3.49 nm after two (2C_B) and three cycles (3C_B), respectively. The PdI decreased from 0.43 to values between 0.24 and 0.31, indicating a more homogeneous population of particles after cycles. The ζ-potential remained negative in 0C and 1C, shifting from −15.90 to −19.33 mV, which is consistent with increased exposure of alginate surface charge as large aggregates were disrupted.

**Figure 5:**
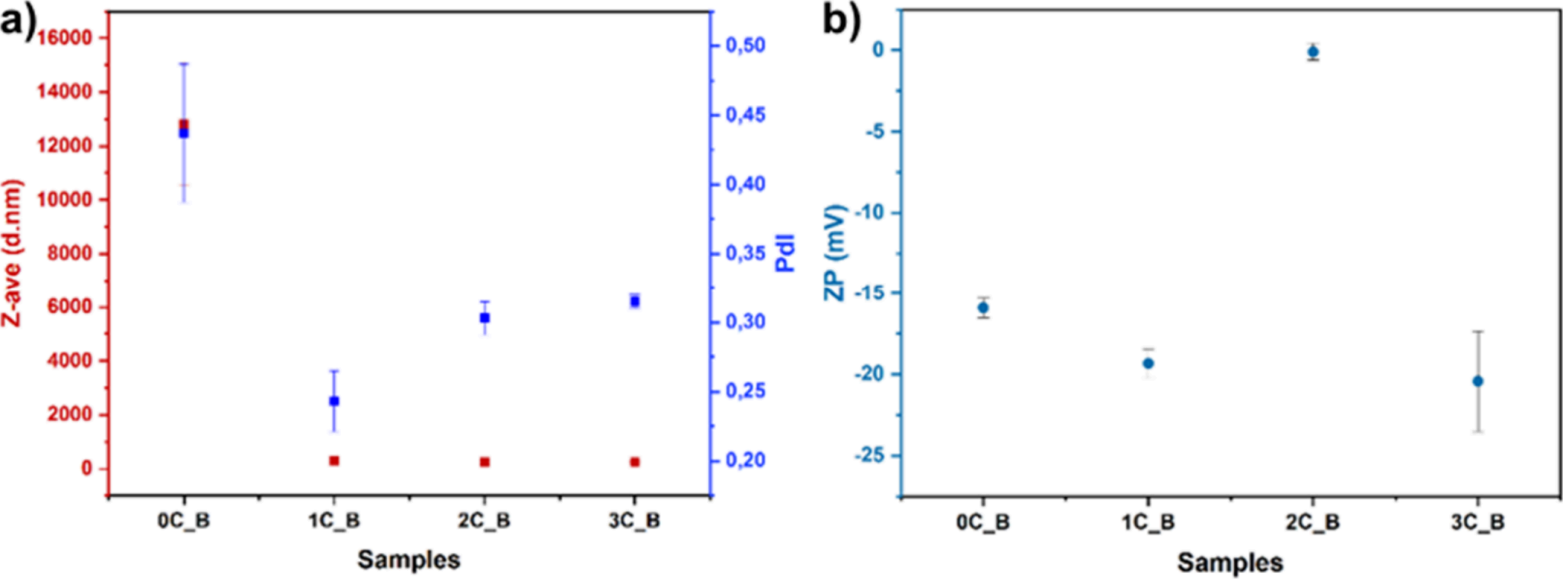
(a) Z-Ave, PDI, and (b) ζ-potential for the 2 L synthesis at 0C_B, 1C_B, 2C_B, and 3C_B.

At 2C, the ζ-potential approached neutrality, around −0.09 mV, indicating a marked reduction in the apparent surface charge. This profile should be interpreted cautiously, since it may be associated with several physicochemical factors, including pH, ionic strength, polysaccharide concentration, Ca^2+^-mediated charge screening, particle association, and the processing conditions applied during high-pressure homogenization. After the third cycle, the ζ-potential returned to approximately −20.43 mV, suggesting a more negative apparent surface charge.^30–32^

The pH of polysaccharide-based colloidal systems is an important physicochemical parameter because it can affect ionization, surface charge, and the measured ζ-potential, together with polymer concentration and ionic strength. Carneiro-da-Cunha et al. showed that pH, ionic strength, and polysaccharide concentration influence both ζ-potential and hydrodynamic diameter in polysaccharide systems.^33^ Therefore, the pH of the main calcium alginate nanoparticle suspensions, including the 0.05% (w/v) formulations (0C_LC to 3C_LC and 0C_B to 3C_B) and the 1% (w/v) formulation (1% PT_B to 1% 3C_B), was measured at room temperature (25 ± 2 °C) after each homogenization cycle. The recorded pH values remained in the alkaline range, approximately between 8.2 and 8.7. Since pH influences the ionization of alginate carboxyl groups and consequently affects surface charge, these results indicate that pH may have contributed to the measured ζ-potential values. However, because the pH remained within a relatively narrow alkaline range, the near-zero ζ-potential observed for the 1% formulation after high-pressure homogenization is more likely related to the combined effects of increased polymer concentration, ionic strength, and Ca^2+^-mediated charge screening.

Together, the progressive size reduction, the lower PdI, and the recovery of a negative ζ-potential after three cycles support the reproducibility of the process at liter scale and the usefulness of HPH as a refining step for calcium alginate nanoparticle suspensions.

### 3.2 Morphological and Elemental Characterization by FEG-SEM, EDS, and SEM

The morphological and elemental characterization of calcium alginate nanoparticles was performed using field emission gun scanning electron microscopy (FEG-SEM), energy-dispersive spectroscopy (EDS), and conventional scanning electron microscopy (SEM). FEG-SEM images, as shown in Figure 6a–d, revealed irregular aggregates in the submicron range, together with discrete nanostructures dispersed within the clusters. Elemental mapping by EDS further confirmed the chemical composition of the nanoparticles. The image (Figure 6d) demonstrated the presence of oxygen, calcium, and sodium as the main elements, while the individual maps in Figure 6e showed a homogeneous distribution of calcium across the alginate matrix. A representative qualitative EDS spectrum is provided in Figure S1, showing the characteristic signals of C, O, Na, and Ca, supporting the elemental mapping presented in Figure 6.

**Figure 6:**
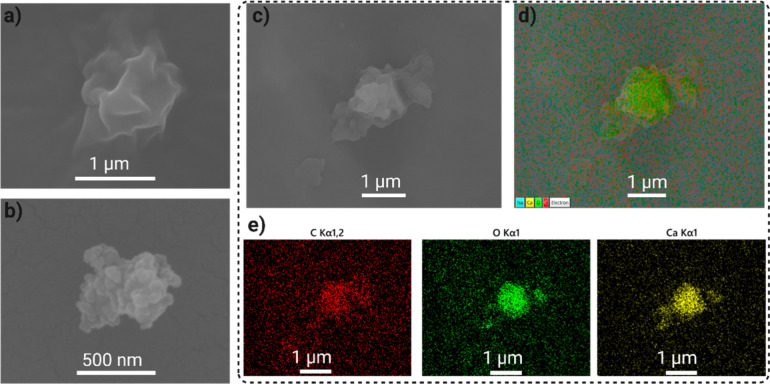
Representative FEG-SEM images and elemental mapping of calcium alginate nanoparticles. Images in (a–c) nanoparticle morphology at different magnifications; (d) presents the EDS elemental mapping overlay and (e) shows qualitative elemental maps for carbon (C), oxygen (O), and calcium (Ca).

The SEM micrographs in Figure 7 show the morphological evolution of calcium alginate nanoparticles produced at the laboratory scale (400 mL) before and after high-pressure homogenization. Images (i) and (ii) correspond to the untreated suspension (0C), where large, compact aggregates and irregular clusters predominate, indicating poor dispersion and strong interparticle association.

**Figure 7:**
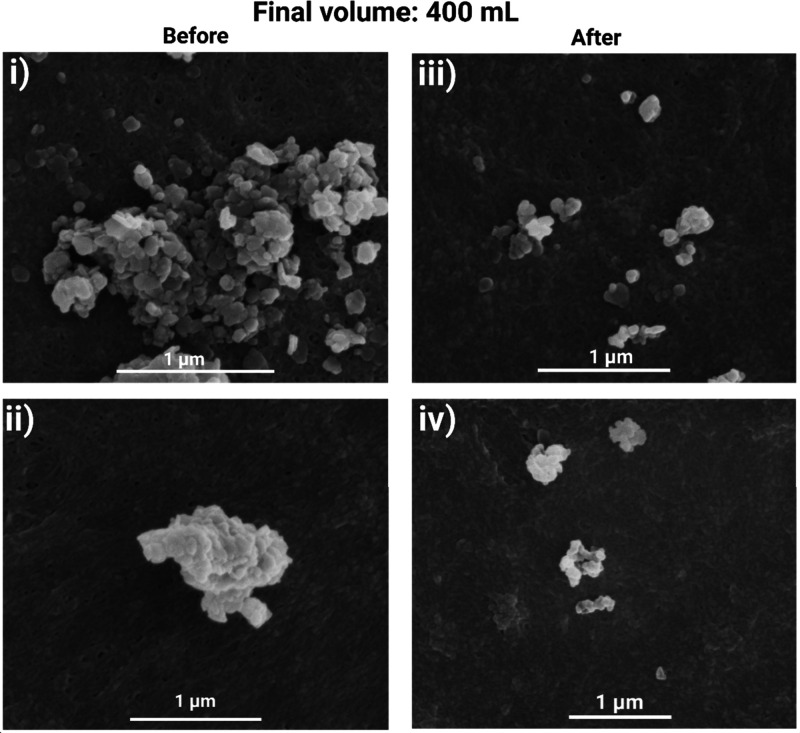
Micrographs show SEM images of calcium alginate nanoparticles obtained at a final volume of 400 mL. Panels (i) and (ii) show clusters of aggregated nanoparticles with irregular morphology before homogenization, while panels (iii) and (iv) show the material after homogenization, with more dispersed nanometric structures and a more homogeneous appearance.

After homogenization, images (iii) and (iv) reveal a substantial reduction in aggregate size, with the presence of smaller nanometric structures showing a more uniform morphology. This progressive morphological refinement demonstrates the efficiency of HPH in disrupting clusters, leading to more uniform and better-dispersed nanoparticles throughout the sample. Together, the FEG-SEM, EDS, and SEM analyses provide complementary insights into the morphology and composition of the nanoparticles.

## 4 L Batch with 1% (w/v) Sodium Alginate and 1% (w/v) CaCl_2_

Previous studies have often employed very low concentrations of sodium alginate, like those used in this work, to obtain nanoparticles.^34^ The hypothesis tested here was whether high-pressure homogenization (HPH) could also reduce aggregates formed at higher concentrations into nanoparticles. This approach is particularly important because producing nanoparticles from more concentrated sodium alginate dispersions would allow for higher batch yields compared to the conventional 0.05% formulations. To further examine how polymer concentration influences nanoparticle formation, a 1% (w/v) sodium alginate solution was first prepared and converted into calcium alginate through ionic gelation, after which the resulting nanosuspension was subjected to high-pressure homogenization (HPH). This step aimed to verify whether HPH could effectively reduce the size of particles produced at higher concentrations, where aggregation was more pronounced. Because micrometer-sized clusters were initially observed, a pretreatment (PT) cycle at 100 bar was applied before the three main homogenization cycles at 1000 bar. This initial stage helped to break down larger aggregates and improve dispersion during the subsequent high-pressure processing. The following results show the variations in hydrodynamic diameter, ζ-potential and polydispersity index after one, two, and three cycles, as illustrated in Figure 8.

**Figure 8:**
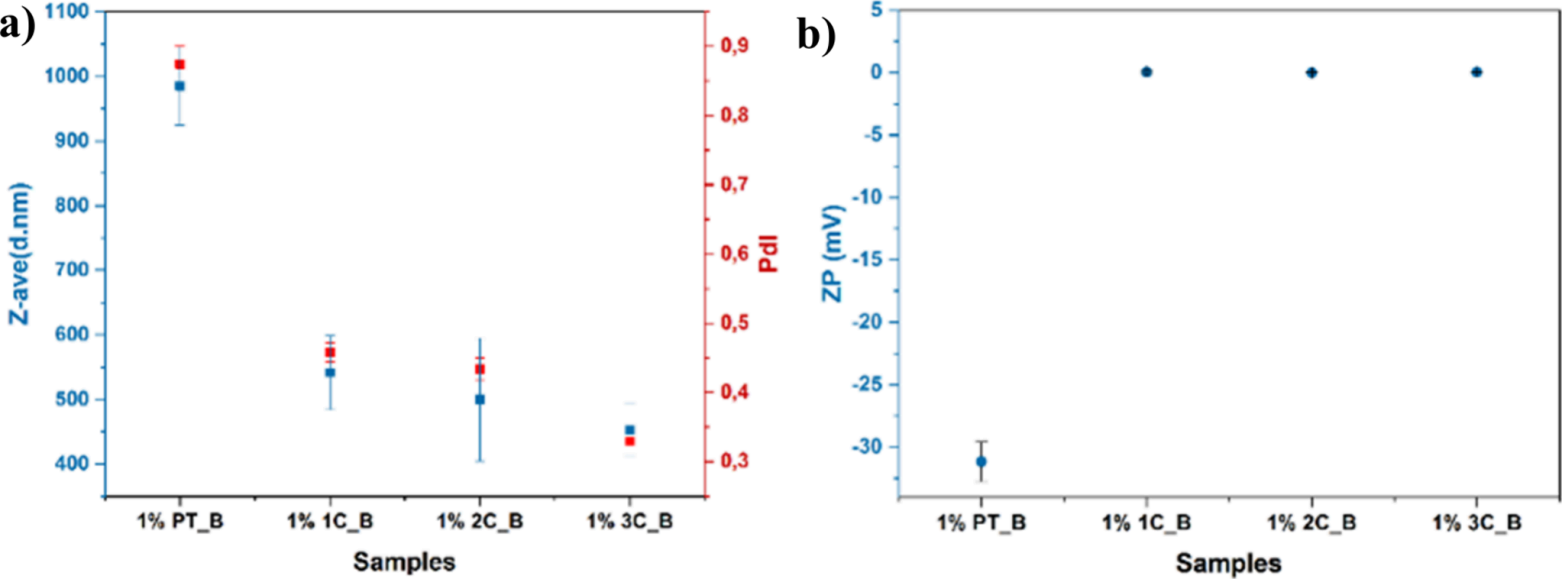
Effects of pretreatment and subsequent high-pressure homogenization cycles on (a) hydrodynamic diameter (Z-Ave) and polydispersity index (PdI); (b) ζ-potential (ZP) of 1% (w/v) sodium alginate suspensions. Data are presented as mean ± standard deviation.

To further evaluate the processing of more concentrated systems at larger batch volume, a 2 L batch was prepared using a higher polymer concentration (1% w/v sodium alginate and 1% w/v CaCl_2_), extending the conditions previously tested at lower concentrations. This experiment was designed to determine whether high-pressure homogenization (HPH) could also refine the larger aggregates typically formed at elevated alginate contents, while maintaining the advantages observed in the smaller scale runs. After ionic gelation under these concentrated conditions, the resulting calcium alginate dispersion exhibited larger and more compact structures in the micrometer range, which is expected when higher alginate levels promote the formation of denser particulate networks.

As expected for concentrated formulations, the initial dispersion contained large micrometer-sized clusters. Therefore, the batch was first subjected to a pretreatment pass at 100 bar (1% PT_B) before the main homogenization sequence at 1000 bar for one, two, and three passes (1% 1C_B, 1% 2C_B, and 1% 3C_B, respectively).

This pretreatment at 100 bar resulted in a mean hydrodynamic diameter of 1019.20 ± 27.20 nm, a PdI of 0.843 ± 0.056, and a ζ-potential of −31.17 ± 1.57 mV, indicating partial stabilization prior to high-pressure homogenization at 1000 bar. Subsequent homogenization further decreased particle size, from 573.03 ± 15.10 nm at 1% 1C_B to 547.13 ± 17.32 nm at 1% 2C_B and 435.23 ± 4.57 nm at 1% 3C_B. PdI values also decreased from 0.429 ± 0.053 to 0.390 ± 0.089 and 0.346 ± 0.038, suggesting a progressive narrowing of the size distribution despite the concentrated formulation. ζ-potential values varied, with 0.0354 ± 0.10 mV at 1% 1C_B, – 0.0033 ± 0.022 mV at 1% 2C_B, and 0.0325 ± 0.013 mV at 1% 3C_B. Taken together, these results demonstrate that the pretreatment at 100 bar followed by the HPH sequence remains effective at larger volumes and higher polymer concentrations. The process successfully converts highly aggregated dispersions into nanoscale structures, confirming its robustness and supporting its suitability for scaled-up nanoparticle production with increased batch yields.

### 4.1 Spray Drying Process and Powder Characterization

Following nanoparticle formation and refinement by high-pressure homogenization, the next step was to convert the aqueous nanosuspension into a dry powder. This stage is essential for evaluating processability, morphological integrity, and long-term stability, as well as for enabling storage, transport, and incorporation into final dosage forms. Moreover, transforming the nanosuspension into a solid-state provides a more complete assessment of the feasibility of the integrated ionic gelation-HPH-drying process for scalable production. The formulation containing 1% (w/v) sodium alginate was selected for spray drying because its higher solid content improved material recovery and favored the formation of a continuous powder phase.

Figure 9a–c summarizes the final stage of the process and its outcome, illustrating the transition from nanosuspension to solid-state calcium alginate nanoparticle powder and its subsequent characterization. Figure 9a shows the collector assembly of the spray-dryer used to dry the nanosuspension, Figure 9b presents the collection flask containing the deposited material, and Figure 9c displays the fine powder obtained after drying. These images demonstrate the effectiveness of the drying process, confirming the successful transition from nanosuspension to solid-state calcium alginate nanoparticle powder.

**Figure 9:**
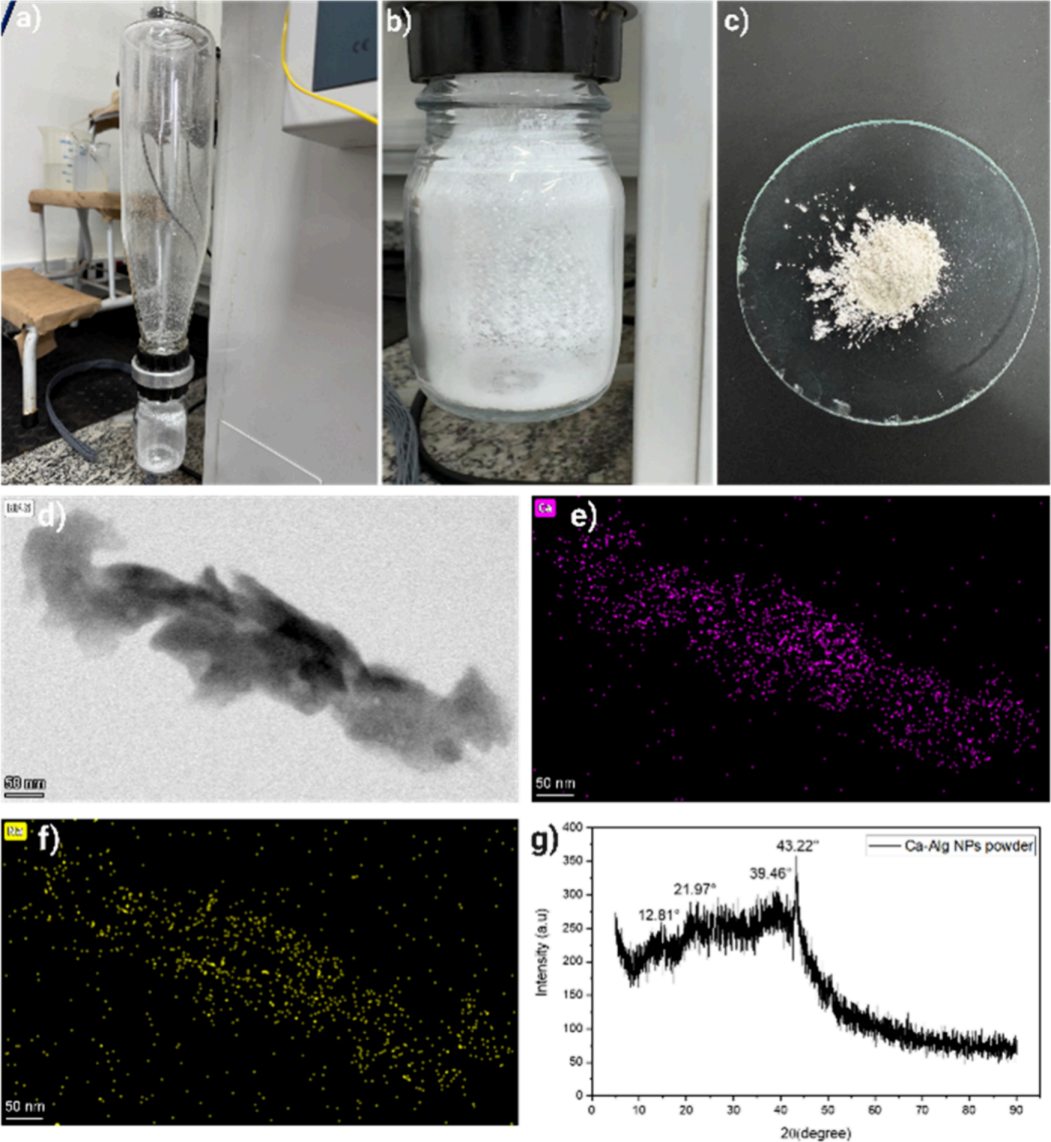
Production and characterization of calcium alginate nanoparticle powder: (a) collector assembly used in the spray-dryer system; (b) collection flask showing the powder deposition after drying; (c) final appearance of the calcium alginate nanoparticle powder; (d) TEM micrograph; (e and f) elemental mapping of Ca and Na; and (g) X-ray diffraction pattern of the Ca-Alg NPs powder.

The spray-drying process resulted in a powder yield of approximately 48%, which is within the range reported in the literature^14,35^ and may be associated with typical product losses during spray drying, particularly particle adhesion to the drying chamber and cyclone surfaces, in addition to the influence of formulation and operating parameters. The recovered powder presented a moisture content of 9.7%. Although this value is slightly above the 7% value reported in the literature for dried powders,^14^ this result may be associated with the possible presence of residual CaCl_2_, whose hygroscopic nature may have contributed to additional moisture retention.

Transmission electron microscopy (TEM) images and the corresponding elemental mappings (Figure 9d–f) revealed nanostructured particles in the dried powder, with apparent sizes ranging from the nanometric scale to a few hundred nanometers and with some degree of particle association. The calcium (Ca) and sodium (Na) elemental maps obtained by energy-dispersive X-ray spectroscopy (EDS) demonstrated a homogeneous distribution of these elements within the nanoparticles, supporting the formation of the calcium alginate matrix. The morphologies observed in the dried nanoparticle powder are further presented in Figure S2 of the Supporting Information. After redispersion, Nanoparticle Tracking Analysis (NTA) was used as a complementary technique to confirm the nanoscale size distribution of the calcium alginate nanoparticles after drying and redispersion. As shown in Figure S3 (Supporting Information), the NTA results corroborate the particle size range obtained by dynamic light scattering, with a mean diameter around 200 nm and a modal size near 150 nm. Representative particle tracking videos are available in Supporting Information (Supplementary Video S1).

The discrepancy between DLS and TEM particle sizes is consistent with the literature and reflects the different principles of these techniques and the different physical states of the samples analyzed. DLS measures the hydrodynamic diameter of particles in suspension, including the solvated polymer layer, and is highly sensitive to aggregates and to a minor fraction of larger particles, especially in concentrated or polydisperse systems. By contrast, TEM evaluates dried particles under vacuum and therefore mainly reflects the size of dehydrated solid structures. Previous studies have shown that DLS values are often larger than TEM values for this reason, and that broad distributions or the presence of larger associated structures may further shift DLS results toward higher diameters.^36–38^ Thus, the predominance of particles below 200 nm in TEM, together with the larger DLS mean diameter for the 1% formulation, suggests that the original concentrated nanosuspension contained hydrated and partially associated nanostructures. This interpretation is supported by the NTA results of the redispersed powder, which showed a mean diameter around 200 nm, closer to the TEM observations. To facilitate visualization of the experimental window investigated in this study, the main formulation parameters, processing conditions, and physicochemical results are summarized in Table S1 in the Supporting Information.

The X-ray fluorescence (XRF) spectrum of the spray-dried calcium alginate powder, presented in Figure S4a,b in the Supporting Information, showed the presence of calcium and chlorine, consistent with the incorporation of CaCl_2_ during ionic cross-linking. Sodium was also detected at lower intensity, indicating partial retention of the original sodium form of the polymer and incomplete ion exchange during gelation. Minor amounts of Fe, Cu, Br and Zn were also detected in the quantitative XRF analysis shown in Figure S4b, each at levels below 0.020%, attributable to the marine origin of the alginate precursor, which naturally retains halogens and metal residues from seawater or the algal cell wall. This elemental profile aligns with previous studies describing the inorganic composition of alginates derived from brown seaweeds.^39,40^

The X-ray diffraction pattern of calcium alginate nanoparticles displayed three weak diffraction peaks at 2θ = 12.81°, 21.97°, and 39.46°, along with a more prominent diffraction peak at 43.22°, as shown in Figure 9g. This pattern reveals a predominantly amorphous structure, consistent with previous studies that reported similar structural organization for calcium alginate particles cross-linked with Ca^2+^ ions.^41^

The presence of diffuse and low-intensity peaks indicates limited long-range order, typical of biopolymeric systems where ionic interactions partially organize the polymer chains while maintaining overall amorphous character. The limited structural ordering of the material arises from the coexistence of amorphous and ordered regions. The ordered domains correspond to the alignment of alginate chains around Ca^2+^ ions introduced through cross-linking, forming localized “egg-box” structures, whereas the amorphous regions result from irregular packing of polymer chains.^42^ The distinct diffraction peak at 43.22° may be attributed to possible contribution from calcium-based residues, such as traces of CaCl_2_ formed during the gelation or drying process.^43^ This mixed structural organization directly affects the physicochemical and functional properties of the nanoparticles, including their stability, interaction with encapsulated molecules, and release behavior. For example, Miles et al.^44^ showed that polymers with larger crystallites exhibited faster drug release because these ordered domains reduce diffusion tortuosity, while more amorphous matrices generated slower and sustained release.

Ionic cross-linking with multivalent cations such as Ca^2+^ increases the elastic modulus and structural stability of alginate networks, as reported by Malektai et al.^45^ In contrast, the amorphous regions confer flexibility and allow molecular diffusion. Zhang et al.^46^ demonstrated that tuning the calcium content modulates permeability in dual-cross-linked hydrogels. These combined structural features give calcium alginate a semicrystalline architecture that balances mechanical strength and diffusional properties, making it suitable for drug delivery, encapsulation of bioactive compounds and ion-exchange applications.

These structural features justify the subsequent physicochemical evaluation of the calcium alginate nanoparticles. Comprehensive analyses are presented in Figure 10a–d, which shows the FTIR spectra of samples (a) and (b), as well as the DSC and TGA profiles in (c) and (d), respectively.

**Figure 10:**
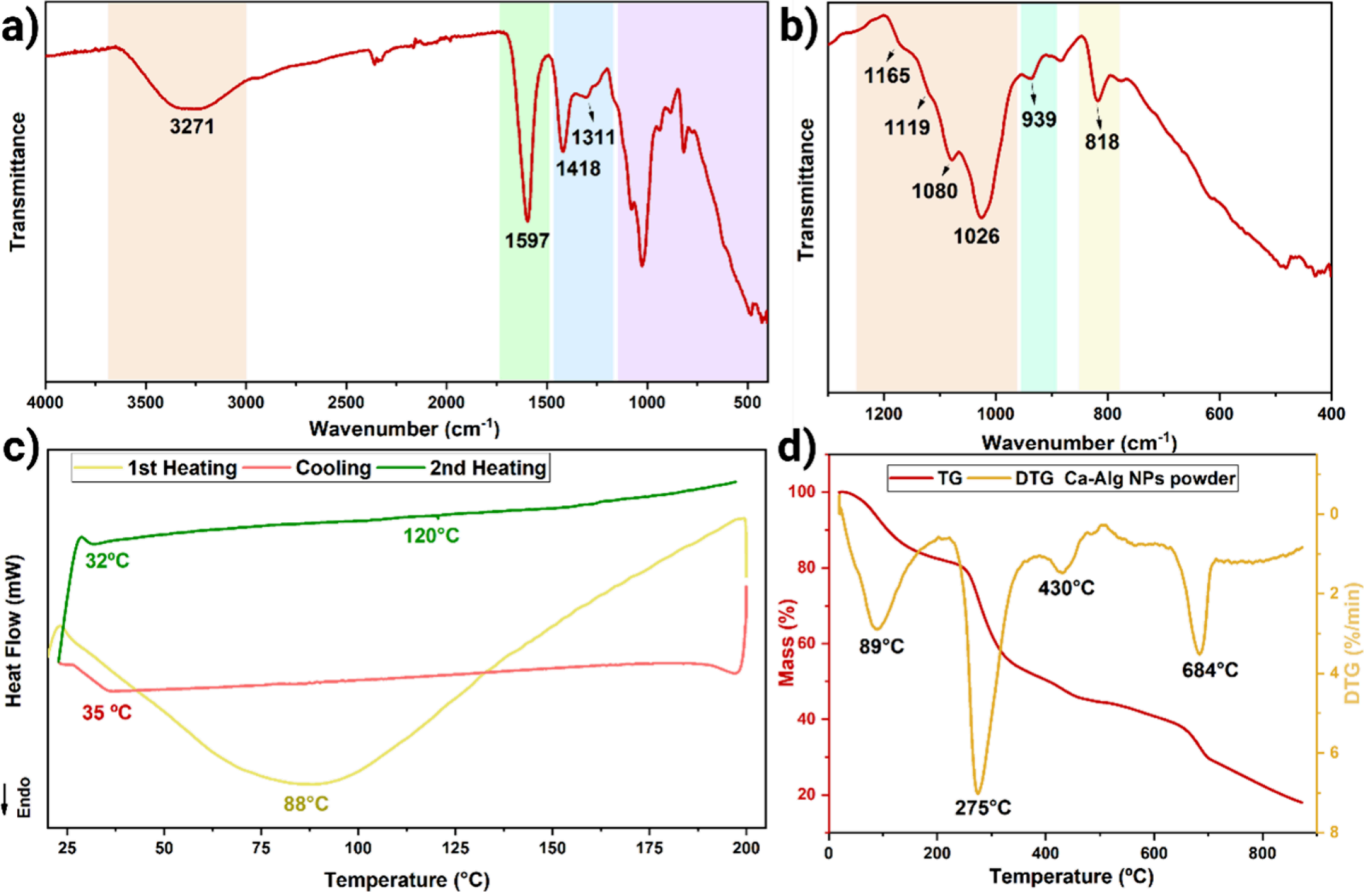
Physicochemical and thermal characterization of calcium alginate nanoparticles. (a) FTIR spectrum recorded in the 4000–400 cm^–1^ range; (b) FTIR spectrum recorded in the 1300–400 cm^–1^ range; (c) DSC thermogram showing the first and second heating and cooling cycles; and (d) TGA/DTG curves.

The broad band at 3271 cm^–1^ corresponds to the O–H stretching vibration of hydroxyl groups and adsorbed water, a typical feature of polysaccharides and reported for both sodium and calcium alginate structures.^41^ The bands observed at 1597 and 1418 cm^–1^ are attributed to the asymmetric and symmetric stretching vibrations of carboxylate groups (COO^–^), respectively. These vibrations are characteristic of alginate salts and are consistent with the coexistence of sodium and calcium alginate phases. Similar carboxylate bands were described by Daemi and Barikani^13^ and Larosa et al.^41^ for calcium alginate nanoparticles and calcium–alginate beads.

In Figure 10b, distinct absorption bands between 1200 and 900 cm^–1^ (1165, 1119, 1080, and 1024 cm^–1^) correspond to C–O–C and C–O stretching vibrations of the pyranose ring, confirming the structural integrity of the polysaccharide backbone, as also reported by Sakugawa et al.^47^ and Lozano-Vázquez et al.^42^ for alginate systems. The band at 939 cm^–1^ is associated with C–C–H and C–O–H deformations within the sugar ring, whereas the weak band at 818 cm^–1^ is related to skeletal vibrations of mannuronic (M) and guluronic (G) units, in agreement with previous assignments for calcium alginate xerogels.^42^

The DSC thermogram (Figure 10c) displays three thermal stages corresponding to the first heating, cooling, and second heating, which characterize the thermal behavior of calcium alginate NPs powder. During the first heating, a broad endothermic event was detected around 88 °C, associated with the evaporation of residual and bound water, which is consistent with the mass loss observed in the TGA curve (Figure 10d). Calcium alginate is a predominantly amorphous copolymer^48^ composed of β-D-mannuronic (M) and α-L-guluronic (G) acid units and therefore does not exhibit melting (*T*_m_) or crystallization (*T*_cc_) transitions typical of crystalline polymers. Its glass transition (*T*_g_) is instead associated with the segmental mobility of the polymer chains and generally occurs near 120 °C for calcium-cross-linked structures,^49^ as seen in the cooling and second heating cycle and more clearly shown in the magnified view presented in Figure S5 of the Supporting Information. Since the DSC curves also showed low-temperature endothermic events, complementary X-ray fluorescence (XRF) analyses were examined, revealing the presence of residual CaCl_2_. This finding suggests that the endothermic events observed near 32 and 35 °C in the second heating cycles are consistent with phase transitions of calcium chloride hydrates rather than intrinsic polymer transitions. Such transitions have been reported for CaCl_2_ hydrates, whose melting and crystallization occur near 30 °C.^50,51^

Altogether, the analytical results support the coexistence of sodium and calcium alginate species, in agreement with the XRF elemental composition, which identified calcium, sodium, and chlorine in the same material. These findings indicate partial ionic exchange between Na^+^ and Ca^2+^ and the formation of a heterogeneous but stable cross-linked alginate network.

Figure 10d shows the thermogravimetric (TG) and derivative thermogravimetric (DTG) curves of calcium alginate nanoparticle powder (NPs Ca-Alg). Four main mass loss events are observed, with peaks at approximately 89, 275, 430, and 684 °C. The first event, ending near 200 °C with a mass loss of around 20%, is attributed to the removal of physically adsorbed and structurally bound water. This initial mass loss is also influenced by the concentration of calcium chloride (CaCl_2_) used during the gelation process, as higher CaCl_2_ concentrations promote stronger cross-linking, reducing water mobility and consequently affecting the amount of water released upon heating.^48^ The second event, between 200 and 380 °C with a peak at 275 °C, corresponds to the main degradation stage related to the breakdown of the alginate polymer backbone. Additional smaller losses at 430 and 684 °C are associated with the decomposition of carbonaceous residues and calcium-based compounds. The residual mass at 872 °C, about 18% of the total, may correspond to calcium oxide (CaO), consistent with thermal degradation behavior reported for calcium alginate systems.^43^

Water activity (aw) is an important indicator of the fraction of free water present in dried particulate systems and is often more informative than total moisture content when evaluating the stability of a powder. As noted by Baldim et al.,^52^ moisture content represents total water, whereas water activity reflects only the water that remains available to interact with the material. In this study, DSC and TGA showed thermal events associated with bound residual moisture, indicating that some water remained tightly associated with the calcium alginate matrix. Despite this, the spray-dried nanoparticles exhibited a water activity of 0.2844, indicating low free water availability in the system. In dried materials, water activity values below 0.5 are generally associated with reduced moisture-related deterioration, since they strongly limit microbial growth.^53,54^ Therefore, the measured value suggests that the drying step was effective in restricting free water and in producing a solid material with favorable characteristics for storage and handling from the standpoint of low free water availability.

In addition, the calcium alginate powder was evaluated by nitrogen adsorption using the BET method. The analysis revealed that the calcium alginate (Ca-Alg) powder had a BET surface area of 2.08 m^2^/g and a Langmuir surface area of 2.71 m^2^/g. The corresponding average pore widths were 26.46 Å for adsorption and 46.21 Å for desorption. Figure S6 of the Supporting Information shows the textural characterization of the material, including (a) the Langmuir surface area plot, (b) the BET surface area plot, (c) the nitrogen adsorption isotherm, and (d) the nitrogen desorption isotherm. These parameters describe a heterogeneous porous network resulting from ionic gelation and drying, where the rearrangement of the polymeric chains during solvent removal defines the final pore distribution. Comparable results were reported by Zhang et al.,^55^ who observed a BET surface area of 0.22 m^2^/g for pure alginate fibers, increasing to 0.45 m^2^/g with the incorporation of SiO_2_ nanoparticles and 0.25 m^2^/g when hydroxyapatite was added. Abutu et al.^56^ also reported a BET surface area of 2.3 m^2^/g for unmodified calcium alginate beads, which increased to 49.84 m^2^/g after modification with Fe_2_O_3_ nanoparticles, due to the creation of additional active sites and a more open structure that enhances molecular interactions. These differences highlight the strong influence of inorganic additives on the textural properties of alginate-based systems. Collectively, the literature demonstrates that the surface characteristics of calcium alginate materials depend heavily on formulation variables such as nanoparticle incorporation, gelation medium, and drying conditions. These parameters determine the balance between porosity and mechanical stability, which are essential for optimizing the functional performance of calcium alginate systems in applications such as adsorption, encapsulation, and controlled release.^57,58^

## 5 Conclusions

This study established a scalable and organic solvent-free route for producing calcium alginate nanoparticle powders by integrating ionic gelation, high-pressure homogenization and spray drying. The process generated homogeneous nanoparticles with controlled size and stable morphology without the use of surfactants or organic solvents. High-pressure homogenization was critical for aggregate disruption and dispersion refinement, while spray drying enabled the formation of a structurally preserved and free-flowing powder. Comprehensive physicochemical and thermal analyses (XRD, XRF, TG/DTG, DSC, and BET) confirmed an amorphous matrix with semicrystalline regions, high calcium content and adequate stability. The results demonstrate the efficiency and reproducibility of the proposed method, particularly for ionic gelation scale-up combined with high-pressure homogenization as a refining step. Overall, this approach provides a simple and environmentally sustainable strategy for obtaining powdered calcium alginate nanoparticles, with potential relevance for future investigation in encapsulation systems and controlled delivery applications.

## Supplementary Material





## Notes

Representative nanoparticle tracking analysis (NTA) video showing the Brownian motion and trajectories of calcium alginate nanoparticles after redispersion of the spray-dried powder in water (WMV)
Additional characterization data, comprising a representative EDS spectrum, TEM images at different magnifications, nanoparticle tracking analysis after powder redispersion, X-ray fluorescence spectra and quantitative elemental analysis, a magnified view of the DSC thermogram in the region around 120 °C, nitrogen adsorption analysis, a representative NTA video, and a summary table of the experimental process window and key physicochemical results (PDF)
