# Supplementary material for: A Simple, Organic Solvent-Free, and Scalable Process for Producing Calcium Alginate Nanoparticle Powder by High-Pressure Homogenization and Spray Drying
Source: ACS Omega. 2026 Jun 29;11(27):40005–17. doi: 10.1021/acsomega.6c01781 (PMC13382728; doi:10.1021/acsomega.6c01781)
Supplement: Supplementary file 2 [file ao6c01781_si_002.pdf]

SUPPORTING INFORMATION

A simple, organic solvent-free, and scalable process for producing calcium alginate nanoparticle powder by high-pressure homogenization and spray drying

Paulo Augusto Marques Chagas[a*], João Otávio Donizette Malafatti[b], Lívia Rodrigues Boueri de Souza[a], Maria Sirlene Morais[a], Gabriela Fávero Galvão[a], Guilherme Alves Pinto[a], Luiz Henrique Capparelli Mattoso[b], Wanderley Pereira de Oliveira[a*]

[a]Faculty of Pharmaceutical Sciences of Ribeirão Preto, University of São Paulo (FCFRP USP), Ribeirão Preto, SP, CEP 14040-903, Brazil

[b]National Laboratory for Nanotechnology for Agriculture (LNNA), Embrapa Instrumentação, Rua XV de Novembro 1452, Centro, São Carlos, SP 13561-206, Brazil

* Corresponding author.

E-mail address: paulochagas@usp.br; wpoliv@usp.br

CONTENTS

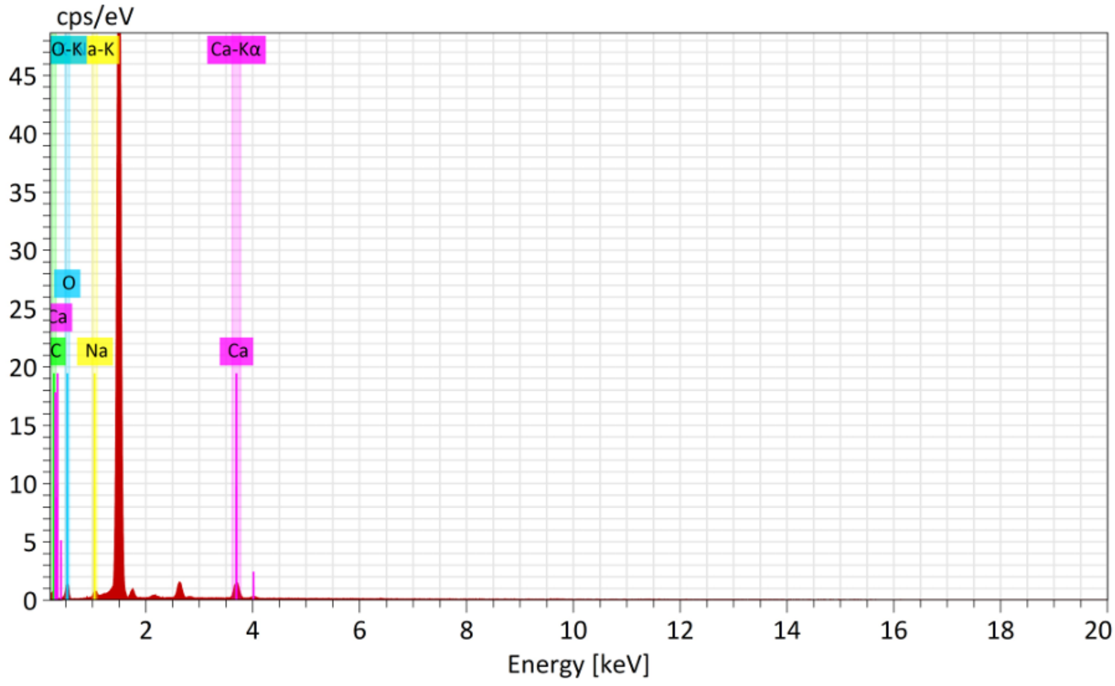

Figure S1: Representative qualitative EDS spectrum of calcium alginate nanoparticles showing the characteristic energy signals of C, O, Na, and Ca.

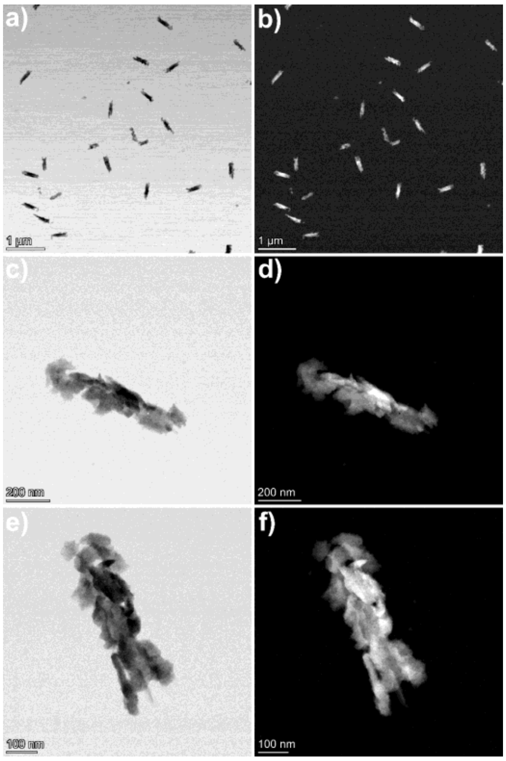

Figure S2: Transmission electron microscopy (TEM) images illustrating the morphology and structure of the nanoparticles at different magnifications. Panels (a, c, e) correspond to bright-field images, and (b, d, f) to dark-field images. Scale bars: 1 µm in (a, b), 200 nm in (c, d), and 100 nm in (e, f).

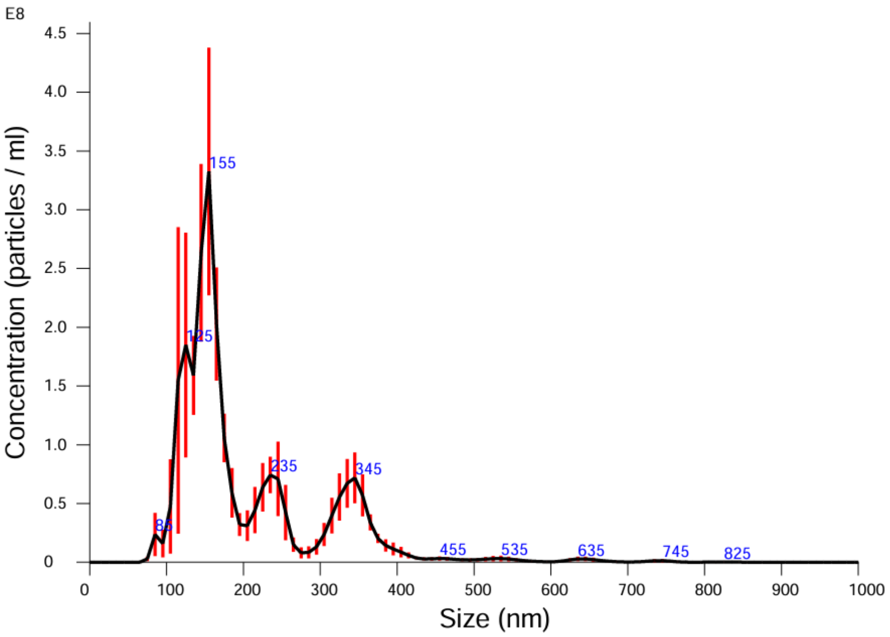

Figure S3: Averaged particle concentration as a function of particle size obtained by Nanoparticle Tracking Analysis (NTA). The curve represents the mean size distribution derived from multiple recordings, while error bars indicate ± one standard error of the mean.

a)

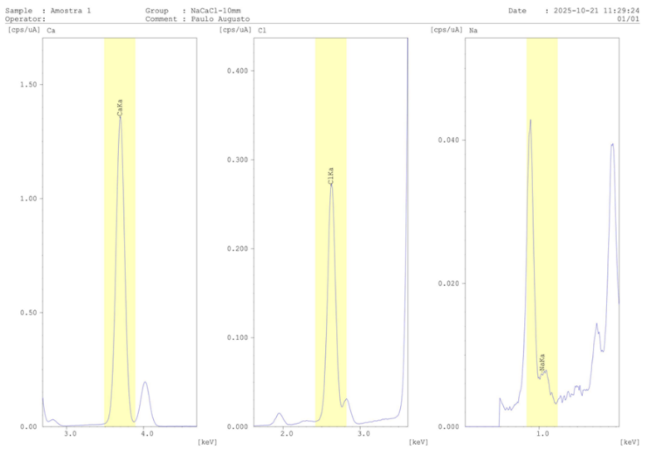

b)

Quantitative Result

Analyte	Result	[3-sigma]	Proc.-Calc.	Line	Int.(cps/uA)
Ca	57.563 %	[0.167]	Quan-FP	CaKa	42.9964
Cl	34.627 %	[0.193]	Quan-FP	ClKa	3.6339
Na	7.295 %	[1.280]	Quan-FP	NaKa	0.0478
S	0.239 %	[0.074]	Quan-FP	S Ka	0.0135
K	0.130 %	[0.028]	Quan-FP	K Ka	0.0258
Si	0.095 %	[0.016]	Quan-FP	SiKa	0.0551
Cu	0.019 %	[0.003]	Quan-FP	CuKa	0.2732
Sr	0.017 %	[0.001]	Quan-FP	SrKa	0.7170
Zn	0.012 %	[0.001]	Quan-FP	ZnKa	0.0084
Fe	0.002 %	[0.004]	Quan-FP	FeKa	0.0059
Br	0.001 %	[0.000]	Quan-FP	BrKa	0.0046

Figure S4: X-ray fluorescence data of the spray-dried calcium alginate powder. a) XRF spectra showing the main signals attributed to Ca, Cl, and Na. b) Quantitative elemental analysis obtained from the XRF measurement.

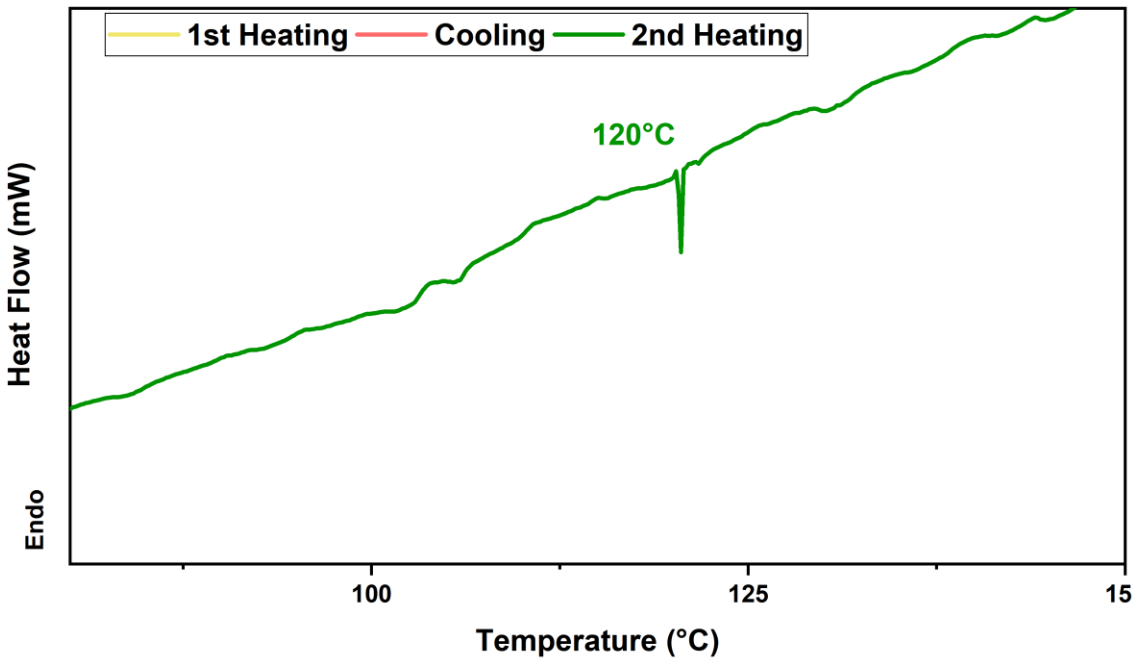

Figure S5: Magnified view of the DSC thermogram of the spray-dried calcium alginate powder in the temperature region around 120 °C.

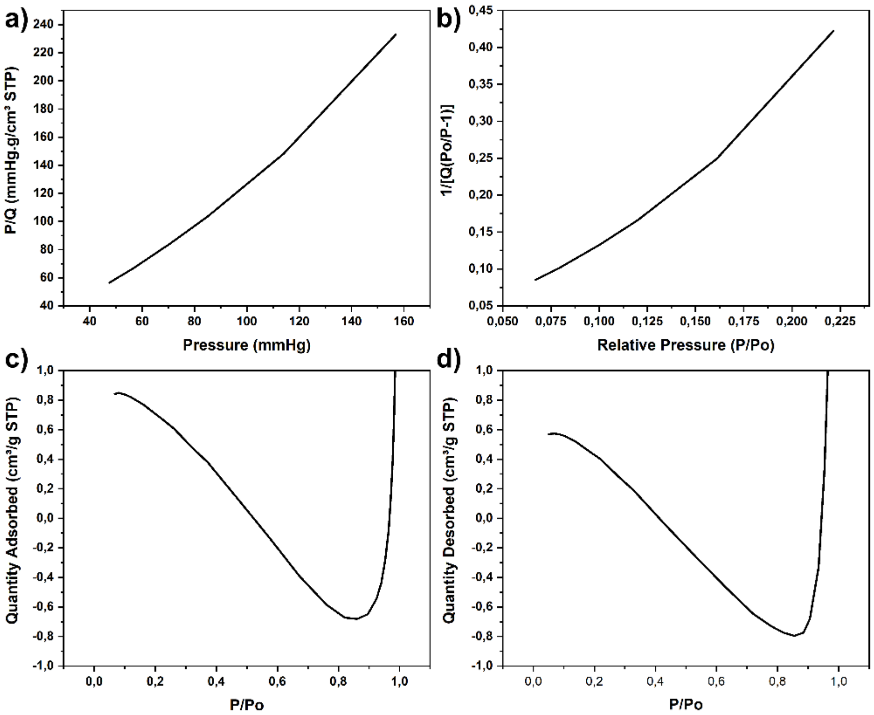

Figure S6: Nitrogen adsorption analysis of the calcium alginate powder at 77 K, (a) Langmuir surface area plot used to calculate the surface area from the linear correlation between pressure and the P/Q term; (b) BET plot showing the linear region applied to determine the BET-specific surface area; (c) nitrogen adsorption isotherm as a function of relative pressure (P/Po); and (d) nitrogen desorption isotherm obtained from the desorption branch.

Calcium alginate nanoparticle powder.wmv

Supplementary Video S1: Representative nanoparticle tracking analysis (NTA) video showing the Brownian motion and trajectories of calcium alginate nanoparticles after redispersion of the spray-dried powder in water.

Table S1: Summary of the process window and key physicochemical outcomes for calcium alginate nanoparticle production by ionic gelation, high-pressure homogenization, and spray drying.

Alginate (% w/v)	Batch volume	$CaCl_2$ condition	HPH condition	Spray-drying condition	Hydrodynamic diameter (nm)	PdI	ζ-potential (mV)	Water activity	Yield (%)	Redispersibility
**0.05**	400 mL	1% (w/v), alginate dripped into $CaCl_2$ at 1100 rpm, 1 mL/min	0C, no HPH	Not applied	1,282.0 ± 229.0	0.44 ± 0.05	-15.9 ± 0.62	ND	ND	ND
**0.05**	400 mL	1% (w/v), same as above	1C, 1000 bar	Not applied	252.9 ± 3.3	0.31 ± 0.03	-9.38 ± 1.69	ND	ND	ND
**0.05**	400 mL	1% (w/v), same as above	2C, 1000 bar	Not applied	232.5 ± 8.4	0.31±0.03	-10.77 ± 0.55	ND	ND	ND
**0.05**	400 mL	1% (w/v), same as above	3C, 1000 bar	Not applied	223.5 ± 5.4	0.37 ± 0.03	-11.97 ± 0.12	ND	ND	ND
**0.05**	2 L	1% (w/v), alginate dripped into $CaCl_2$ at 1100 rpm, 1 mL/min	0C, no HPH	Not applied	12,880.0 ± 1,890.0	0.437 ± 0.05	-15.90 ± 0.62	ND	ND	ND
**0.05**	2 L	1% (w/v), same as above	1C, 1000 bar	Not applied	296.06 ± 1.53	0.243 ± 0.022	-19.33 ± 0.9	ND	ND	ND
**0.05**	2 L	1% (w/v), same as above	2C, 1000 bar	Not applied	248.47 ± 2.1	0.303 ± 0.012	-0.092 ± 0.52	ND	ND	ND
**0.05**	2 L	1% (w/v), same as above	3C, 1000 bar	Not applied	236.23 ± 3.49	0.315 ± 0.005	-20.43 ± 3.1	ND	ND	ND
**1.0**	2 L	1% (w/v), alginate dripped into $CaCl_2$ at 1100 rpm, 1 mL/min	PT, 100 bar	Not applied	1,019.20 ± 27.20	0.843 ± 0.056	-31.17 ± 1.57	ND	ND	ND
**1.0**	2 L	1% (w/v), same as above	1C, 1000 bar	Not applied	573.03 ± 15.10	0.429 ± 0.053	0.0354 ± 0.10	ND	ND	ND
**1.0**	2 L	1% (w/v), same as above	2C, 1000 bar	Not applied	547.13 ± 17.32	0.390 ± 0.089	-0.0033 ± 0.022	ND	ND	ND
**1.0**	2 L	1% (w/v), same as above	3C, 1000 bar	Feed rate 7 mL/min; atomization pressure 2 kgf/cm²; air flow 20 L/min; nozzle 1 mm; inlet 150 °C; outlet 70 to 80 °C	435.23 ± 4.57 before drying; around 200 after redispersion by NTA	0.346 ± 0.038 before drying	0.0325 ± 0.013 before drying	0.2844	about 48	Powder successfully redispersed; NTA mean diameter around 200 nm and modal size near 150 nm

ND, not determined in the present study. Redispersibility was inferred from the successful resuspension of the spray-dried powder in ultrapure water followed by NTA analysis, which showed a mean particle diameter around 200 nm and a modal size near 150 nm.
